# Longitudinal monitoring of mRNA-vaccine-induced immunity against SARS-CoV-2

**DOI:** 10.3389/fimmu.2023.1066123

**Published:** 2023-01-19

**Authors:** Werner O. Monzon-Posadas, Jasmin Zorn, Kathrin Peters, Maximilian Baum, Hannah Proksch, Celina Beta Schlüter, Tanja Menting, Jernej Pušnik, Hendrik Streeck

**Affiliations:** ^1^ Institute of Virology, University Hospital Bonn, Bonn, Germany; ^2^ German Center for Infection Research (DZIF), Partner Site Bonn-Cologne, Braunschweig, Germany; ^3^ Occupational Medicine Department, University Hospital Bonn, Bonn, Germany

**Keywords:** SARS-CoV-2, COVID-19, B cell, T cell, antibody, vaccination, booster, longitudinal

## Abstract

**Background:**

Worldwide vaccination campaigns significantly reduced mortality caused by SARS-CoV-2 infection and diminished the devastating effects of the pandemic. The first approved vaccines are based on novel mRNA technology and elicit potent immune responses offering high levels of protection from severe disease.

**Methods:**

Here we longitudinally assessed adaptive immune responses during a 12-month follow-up period after the initial immunization with 2 doses of mRNA vaccines and after the booster dose in blood and saliva.

**Results:**

Our findings demonstrate a rapid waning of the anti-spike IgG titers between months 3 and 6 after the initial vaccination (1.7- and 2.5-fold decrease in plasma and saliva, respectively; P<0.0001). Conversely, the frequency of spike-specific memory B cells increased during this period (2.4-fold increase; P<0.0001) while the frequency of spike-specific CD4+ and CD8+ T cells remained stable for all assessed functions: cytotoxicity, IFNγ, IL-2, and TNFα expression. Booster vaccination significantly improved the antibody response in plasma and saliva, with the most profound changes observed in the neutralization capacity against the currently circulating omicron variant (25.6-fold increase; P<0.0001). The positive effect of booster vaccination was also evident for spike-specific IgG+ memory B cell (2.4-fold increase; P<0.0001) and cytotoxic CD4+ and CD8+ T cell responses (1.7- and 1.9-fold increase respectively; P<0.05).

**Conclusions:**

Collectively, our findings offer a detailed insight into the kinetics of adaptive immune response following SARS-CoV-2 vaccination and underline the beneficial effects of a booster vaccination.

## Introduction

The vaccination campaign against the SARS-CoV-2 infection was launched at the beginning of 2021 with the hope to dampen the devastating effects of pandemics by reducing transmission and mortality caused by COVID-19. Remarkably, the first two vaccines to receive approval for use were based on a novel mRNA technology that had not been previously applied in any of the marketed vaccines.

The BNT162b2 and mRNA-1273 vaccines demonstrated an outstanding efficacy of over 90% reduction in severe COVID-19 cases and proved safe for use (1, 2). The high efficacy was due to the robust immune response elicited by the 2-dose full vaccination regimen (1–4). Several studies have confirmed that mRNA vaccines induce the production of neutralizing antibodies, with titers well above those induced by the natural infection (4–6). Furthermore, the mRNA vaccines were effective at triggering the formation of memory B cell and T cell responses (7, 8). These features of mRNA vaccines raised high hopes for the worldwide SARS-CoV-2 vaccination campaign to curb the ongoing pandemic. Unfortunately, however, the virus mutated more rapidly and new variants emerged that would overcome vaccine-induced immunity (9–12). The wild-type virus was consecutively succeeded by the alpha, delta, and omicron variants accumulating mutations in the spike protein (13). The currently circulating omicron variant has more than 30 amino-acid changes in the spike protein, compared to alpha and delta variants where typically less than 15 amino-acid are observed (14). This substantial increase in the antigenic distance from the wild-type spike of vaccines leads to efficient immune evasion and higher transmission rates even in countries with high vaccination rates or natural immunity (15). Furthermore, mutations influenced the replication biology of the variants. Particularly, changes near the furin-cleavage site have been suggested to be associated with enhanced cell entry and increased transmissibility (16, 17).

In addition, it has soon become clear that the immune response induced by vaccines, although initially very potent, rapidly wanes with time after vaccination opening a window of opportunity for breakthrough infections (18). Numerous studies have demonstrated the declining antibody titers with time after the initial vaccination and increased rates of breakthrough infections (13, 19, 20) making it evident that the 2-dose vaccination regimen will not be sufficient for long-term protection from COVID-19 and that booster vaccinations will be necessary to keep immunity at high levels (18, 21). At the beginning of fall 2021, the third dose of mRNA vaccines was approved and recommended firstly for high-risk groups and subsequently for the general population. The booster vaccination initially restored vaccine efficacy by triggering a potent recall immune response (22), but similarly waned rather rapidly (23).

As of November 2022, the vaccine coverage in Germany is 78% for at least one dose, 76% for two, and 62% for three vaccine doses (24). Given that the majority of the population has been vaccinated and that the vaccine-induced antibody titers decline with time, it is important to investigate the persistence of the adaptive immune response following vaccination as this may give critical insights into future vaccination and booster strategies.

Here we performed a comprehensive longitudinal assessment of the adaptive immune response to the initial two doses but also a booster dose of mRNA vaccine in healthy SARS-CoV-2 naïve individuals. Our findings demonstrate the waning of the immune response following vaccination and emphasize the beneficial effect of a booster vaccination.

## Materials and methods

### Study cohort

A total of 77 individuals that were initially vaccinated with 2 doses of mRNA-based SARS-CoV-2 vaccine and 9 months later received a booster vaccination were recruited for the study. All individuals were SARS-CoV-2 naïve at the beginning of the study. Breakthrough infections were monitored by RT-PCR and anti-nucleocapsid ELISA as a part of routine screening at the diagnostics department of the Institute of Virology, University Hospital Bonn. Individuals that contracted an infection were excluded from the findings described in this paper. All participants were either employed or studied at the University of Bonn at the time of sampling but were not necessarily healthcare workers.

### Ethics approval

All participants provided written informed consent approved by the Ethics Committee of the Medical Faculty of the University of Bonn (ethics approval numbers 125/21).

### Sample collection and storage

Study participants provided peripheral blood specimens, saliva, and pharyngeal swabs. Blood was centrifuged and EDTA-plasma was stored until analysis (-80°C). Before the saliva collection participants were instructed not to eat or drink for at least 60 min. Participants were than advised to retain saliva for 1-2 min and expectorate it in a centrifuge tube. Saliva samples were centrifuged to remove solid particles and frozen at -20°C. PBMC were isolated by density gradient centrifugation and cryopreserved in liquid nitrogen.

### 2 Determination of SARS-CoV-S1-specific IgG in plasma

S1-specific IgG titers were determined using an in-house quantitative ELISA. Therefore, microtiter plates with high binding capacity were coated with 100 µl of coating buffer (carbonate-bicarbonate buffer, pH=9.6) containing 1 µg/ml of recombinant S1 domain of the SARS-CoV-2 spike protein (Biotinylated SARS-CoV-2 (COVID-19) S1 protein, Acrobiolabs). Plates were subsequently sealed and incubated overnight at 4°C. Coated plates were washed with wash buffer (PBS with 0.05% (v/v) Tween^®^-20) and blocked (PBS containing 1% (w/v) BSA) to prevent unspecific binding. Cryopreserved EDTA plasma samples were thawed at room temperature and diluted at previously optimized dilution of 1:3200 in a blocking buffer. After blocking plates were washed, incubated with plasma samples and standards (serially diluted pooled plasma of vaccinated individuals), washed again, and incubated with 100 µl HRP-conjugated anti-IgG antibody (Goat anti-Human IgG (Heavy chain) Secondary Antibody, HRP, Invitrogen) diluted 1:8000 in wash buffer. If not stated differently, incubation steps were performed for 1 hour at 37°C. Finally, plates were washed and 100 µl of the substrate solution (TMB Chromogen Solution, Life technologies) was added. The reaction developed at room temperature for 5 min until the addition of 50 µl of 0.2 M H_2_SO_4_. Optical density at 450 nm was measured immediately after the reaction was stopped. The background-subtracted OD_450_ readings were interpolated onto the standard dilution curve that had previously been calibrated to the international WHO standard (NIBSC reference number: 20/136). To determine the positivity cutoff we measured plasma samples from 30 individuals that have never been exposed to SARS-CoV-2. Based on the measurements we determined the cutoff (mean+2xSD) from which the results were considered positive for anti-S1 IgG; 19.2 BAU/mL.

### Determination of SARS-CoV-2 S1-specific IgG and IgA in saliva

The titers of S1-specific IgA and IgG in saliva were measured by in-house quantitative ELISA. Therefore, high-binding microtiter plates were coated with 100 µl of coating buffer (carbonate-bicarbonate buffer, pH=9.6) containing 1 µg/ml of recombinant SARS-CoV-2 S1 protein (Biotinylated SARS-CoV-2 (COVID-19) S1 protein, Acrobiolabs). Following overnight incubation at 4°C, the plates were washed (PBS with 0.05% (v/v) Tween^®^-20), blocked (PBS containing 3% (w/v) BSA), and washed again. Saliva samples were thawed, diluted 1:16 in sample buffer (PBS containing 1% (w/v) BSA), and applied onto S1-coated plates. Blocking and incubation with saliva samples and standard dilutions were performed at 37°C for 1 hour. Subsequently, plates were washed and incubated at 37°C for 1 hour with 100 µl HRP-conjugated anti-IgG antibody (Goat anti-Human IgG (Heavy chain) Secondary Antibody, HRP, Invitrogen) diluted 1:8000 in wash buffer or 100 µl HRP-conjugated anti-IgA antibody (Goat anti-Human IgA (Heavy chain) Secondary Antibody, HRP, Invitrogen) diluted 1:1000 in wash buffer. After the incubation with secondary antibodies, plates were washed and 100 µl of the substrate (TMB ELISA Substrate, High Sensitivity, Abcam) was added. The reaction was stopped by the addition of 100 µl of 1 M H_2_SO_4_ after developing for 5 min at room temperature. Optical density at 450 nm was measured immediately after the addition of the stop solution. The background-subtracted OD_450_ readings were interpolated to the standard dilution curve, derived from measurements of serially diluted highly positive saliva samples, to obtain concentration units relative to the standard (arbitrary units indicated as a.u.). These units are not comparable to those of the plasma ELISA. To determine the positivity cutoff we measured saliva samples from 24 individuals that were seronegative for anti- SARS-CoV-2-spike IgG. Based on the measurements we determined the cutoff (mean+2xSD) from which the results were considered positive; 0.014 a.u. for IgG and 0.012 a.u. for IgA. The same concentrations of S1-specific monoclonal antibody (anti-SARS-CoV-2-RBD antibody, clone CR3022, Abcam) with IgA or IgG constant region was measured to make the OD_450_ readings comparable between the IgG and IgA assays.

### Plaque reduction neutralization assay

The plasma neutralization capacity was determined by a plaque reduction neutralization assay as previously described (25). Briefly, plasma was heat-inactivated and serially two-fold diluted starting with 2-fold up to 32768-fold dilution. Each dilution was combined with 80 plaque-forming units of SARS-CoV-2 (either wild-type, delta, or omicron variant). The inoculum was then added to Vero E6 cells. After the incubation, the inoculum was removed, and cells were overlaid with carboxymethylcellulose-containing media. After 3 days, plates were fixed and stained with crystal violet solution revealing the formation of plaques. The number of plaques was plotted against the serum dilutions, and IC_50_ was determined using the GraphPad Prism software.

### Immunomagnetic isolation of B cells

B cells were enriched from cryopreserved PBMC samples by positive immunomagnetic isolation (Human CD19 MultiSort Kit, Miltenyi Biotec) following the manufacturer’s instructions. Briefly, thawed, and rested PBMCs were resuspended in recommended isolation buffer and labeled with anti-CD19 antibodies coupled to magnetic beads. Bead-labeled cells were then immobilized onto a magnetic column. The column was washed and the flow-through containing B-cell-depleted PBMC was set aside for the assessment of T-cell responses. The column was removed from the magnetic field and immobilized B cells were washed out. To remove the magnetic beads and anti-CD19 antibodies, B cells were treated with enzymes disintegrating the immunomagnetic complexes.

### Detection of S1-specific memory B cells by flow cytometry

Antigen-specific B cells were identified by immunofluorescent tagging with recombinant wild-type SARS-CoV-2 S1 protein, as previously described (25). Briefly, the cells were incubated with the recombinant S1 protein conjugated to two different fluorophores, stained for viability, and subsequently incubated with a mixture of fluorescently labeled antibodies binding surface antigens. Labeled cells were acquired on a flow cytometer (BD FACS Celesta). The frequency of S-specific memory B cells was calculated by subtracting the average frequency of S1-binding memory B cells in healthy donor samples collected before the outbreak of SARS-CoV-2.

### *Ex vivo* stimulation of T cells

B-cell-depleted PBMC fractions were seeded in 96-well U bottom plates and stimulated with wild-type SARS-CoV-2 PepTivator (Miltenyi Biotec) overlapping peptide pools spanning the entire sequence of spike (S) protein, in presence of anti-CD107a-APC (clone H4A3; Biolegend) antibody. One million cells were stimulated per condition, and the final concentration of each peptide in the stimulation mix was 1 µg/ml. As a co-stimulatory signal, antibodies binding CD28 and CD49d (BD FastImmune™ CD28/CD49d) were added to a final concentration of 1 µg/ml. Stimulation was performed at 37°C for a total of 6 hours. As a negative control, an equally treated DMSO-stimulated sample was included for each biological replicate. As positive control cells stimulated with PMA (20 ng/ml) and ionomycin (1 μg/ml) were used. One hour into stimulation, Golgi Stop and Golgi Plug (BD Bioscience) were added (final concentration 1 µg/ml) to inhibit vesicular transport and prevent the secretion of the cytokines from cells.

### Detection of SARS-CoV-2-specific T cells by flow cytometry

Stimulated cells were washed with PBS, and stained with Zombie Aqua (Biolegend) dye for 15 min at 4°C to discriminate viable cells. Subsequently, samples were washed with FACS buffer (PBS supplemented with 2% FCS, 2 mM EDTA, and 0.05% NaN_3_), fixed, and permeabilized in CytoFix/CytoPerm Solution (BD Bioscience) for 15 min at 4°C. Cells were then washed with 1x Perm/Wash Buffer (BD Bioscience), and stained for intracellular markers for 15 min at 4°C using the following antibody conjugates; anti-CD3-APC-Cy7 (clone UCHT1; Biolegend), anti-CD4-BV786 (clone SK3; BD Bioscience), anti-IFNγ-PE (clone B27; Biolegend), anti-TNFα-BV421 (clone Mab11; Biolegend), and anti-IL2-AF488 (clone MQ1-17H12; Biolegend). Labeled cells were then washed with PBS and acquired on FACS Celesta (BD Bioscience). Frequencies of antigen-specific CD4+ T cells were calculated as negative-control-subtracted data. Possible longitudinal fluctuations in laser intensity were monitored and adjusted before each experiment using fluorescent beads (Rainbow beads, Biolegend). The data were analyzed with the FlowJo Software version 10.0.7 (TreeStar).

### Statistical analysis

Statistical analysis was performed using R software (26). Differences between the groups were assessed using the Wilcoxon test for matched data with Holm’s correction for multiple testing. The strength of correlations was evaluated by Spearman’s test. Statistical significance is indicated by the following annotations: *p<0.05, **p<0.01, ***p<0.001, ****p<0.0001.

## Results

### Study design

**Figure 1:**
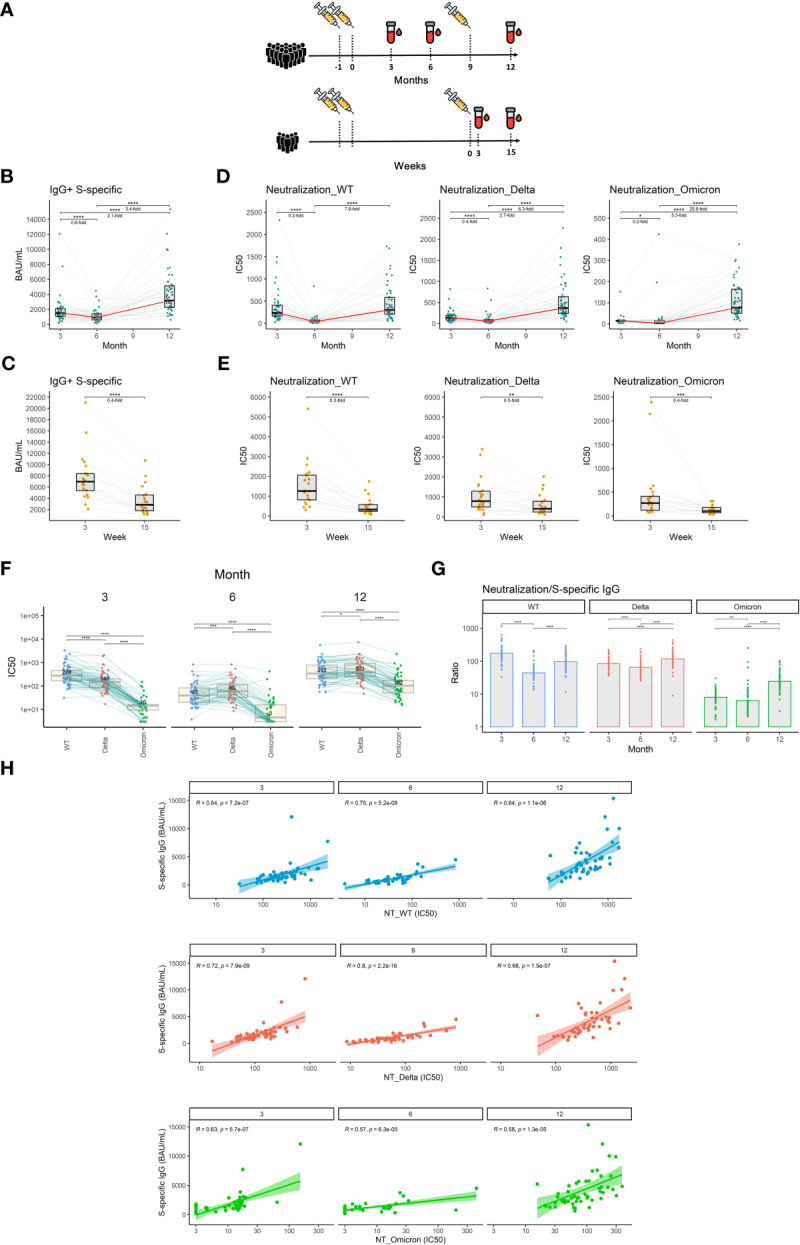
The dynamics of S1-specific antibody response in plasma after the initial and booster doses of mRNA vaccine. **(A)** Timelines demonstrating temporal relationships between vaccination and sampling events of the study participants. **(B)** S1-specific IgG levels in international units (BAU/mL) measured at different time points after the initial vaccination. **(C)** S1-specific IgG levels in international units measured 3 and 15 weeks after the booster vaccination. **(D)** Plasma neutralization capacity measured against the wild-type, delta, and omicron variants for months 3, 6, and 12 after the initial vaccination. **(E)** Plasma neutralization capacity measured against the wild-type, delta, and omicron variants for weeks 3 and 15 after the booster vaccination. The red line connects the median values of each time point. Fold change was calculated as a ratio of the medians of compared time points. **(F)** Comparison of neutralization susceptibility of SARS-CoV-2 variants for months 3, 6, and 12. Median values are given within the boxplots. **(G)** Ratio between the neutralization capacity and S1-specific IgG titers for different time points and different SARS-CoV-2 variants. **(H)** Correlation between plasma levels of S1-specific IgG and plasma neutralization capacity for wild-type, delta, and omicron variants. Differences between the groups were assessed using the Wilcoxon test for matched data. Correction for multiple testing was performed using Holm’s method. The strength of correlations was assessed by Spearman’s correlation test. *p<0.05, **p<0.01, ***p<0.001, ****p<0.0001.

To assess the longevity of the adaptive immune response following SARS-CoV-2 vaccination we monitored antibody, memory B cell, and memory T cell levels of 50 vaccinated individuals for 12 months after the full vaccination with two doses of mRNA vaccine. Samples were taken 3, 6, and 12 months after full vaccination. All individuals received booster vaccination about 9 months after the initial immunization. Individuals with breakthrough infections were excluded from the analysis. Additionally, a group of 20 individuals was recruited to follow the dynamics of immune response immediately after booster vaccination. These individuals were sampled 3 and 15 weeks post-immunization and received the 3 vaccine doses at the same time as the other 50 study participants (**Figure 1A**).

### Vaccination-induced antibodies wane over time

Neutralizing antibodies are the primary antiviral mechanism induced by vaccination and the best-defined correlate of protection against SARS-CoV-2 infection (27). We, therefore, measured plasma levels of IgG specific for the S1 subunit of the SARS-CoV-2 spike protein using an in-house ELISA calibrated to the international WHO standard (NIBSC reference number: 20/136). Our findings indicate that the level of S1-specific IgG declines by 1.7-fold between months 3 and 6 after vaccination with 2 doses of mRNA vaccine (P<0.0001) and then increases by 3.4-fold following booster vaccination between months 6 and 12 (P<0.0001) (**Figure 1B**). Between weeks 3 and 15 after the booster shot, the level of S1-specific IgG decreased 2.5-fold (P<0.0001) (**Figure 1C**). To assess the dynamics of exclusively neutralizing antibodies, we next performed plaque reduction neutralization assays using live un-manipulated SARS-CoV-2 isolates (wild-type, delta, and omicron variants). The neutralization capacity against the wild-type virus declined 5-fold during months 3 and 6 (P<0.0001). In the case of delta and omicron variants, the reductions in neutralizing potency were 2.5- and 5-fold respectively (P<0.0001, P<0.05). On month 12, roughly 3 months following the booster vaccination, the neutralization levels rose 7.6-fold, 6.3-fold, and 25.6-fold for wild-type, delta, and omicron respectively when compared to month 6 (P<0.0001, P<0.0001, P<0.0001) (**Figure 1D**). Furthermore, we observed a 3.3-fold decrease in neutralization capacity against the wild-type virus (P<0.0001), a 2-fold decrease in neutralization capacity against the delta (P<0.01), and a 2.5-fold decrease in neutralization capacity against the omicron variant (P<0.001) between weeks 3 and 15 after the booster vaccination (**Figure 1E**). To demonstrate the different susceptibilities of SARS-CoV-2 variants to neutralization, we next directly compared the plasma neutralization capacity of wild-type, delta, and omicron variants at months 3, 6, and 12 after full 2-dose vaccination. At the first time point, the ancestral variant was most susceptible to neutralization, followed by the delta variant, while omicron showed to be notably more resistant. Interestingly, at month 6, the plasma of vaccinated individuals more efficiently neutralized the delta variant than the wild-type. Omicron remained the most resistant to neutralization at all time points, however, its susceptibility to neutralization considerably increased after booster vaccination (**Figure 1F**). We next compared the ratio between the plasma neutralization capacity and S1-specific IgG titers for all three variants and time points. The data suggest a decreased proportion of neutralizing antibodies against all three variants for month 6 when compared to months 3 and 12. In the case of delta and omicron variants, the highest proportion of neutralizing antibodies was observed after the booster vaccination on month 12 (**Figure 1G**). High S1-specific IgG titers translated well into higher neutralization capacity since we observed strong correlations between the two parameters for all three variants and time points. The correlations were slightly weaker in the case of the omicron variant (**Figure 1H**).

Taken together, our findings demonstrate a waning of the initial antibody response against SARS-CoV-2 after the 2 initial doses but also after the third booster shot of the mRNA vaccine. Importantly, booster vaccination improved the potency of antibody response against the SARS-CoV-2 variants compared to 2 vaccine doses only.

### Booster vaccination augments SARS-CoV-2-specific antibody levels in saliva

As a respiratory virus, SARS-CoV-2 initially infects the upper respiratory tract (28) and infection might be prevented by the presence of antibodies in the upper mucosa, mucus, and saliva.

**Figure 2:**
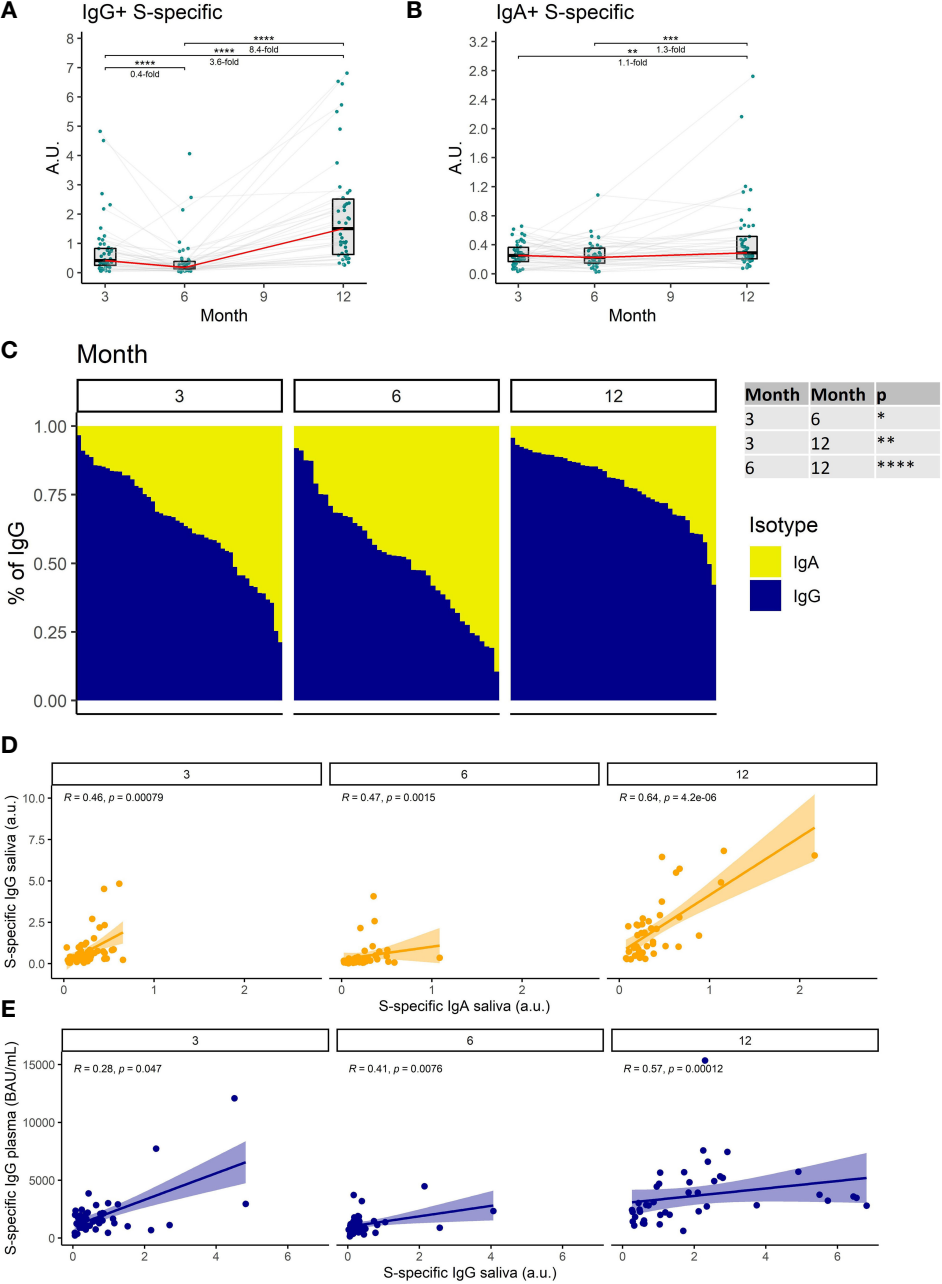
The dynamics of S1-specific antibody response in saliva after the initial and booster doses of mRNA vaccine. S1-specific **(A)** IgG, **(B)** IgA, levels in the saliva of vaccinated individuals for months 3, 6, and 12 after the initial vaccination. The red line connects the median values of each time point. Fold change was calculated as a ratio of the medians of compared time points. **(C)** Relative proportions of IgA and IgG isotypes among the S1-specific antibodies in the saliva of vaccinated individuals. **(D)** Correlations between the salivary S1-specific IgG and IgA for different time points. **(E)** Correlations between the plasma and salivary S1-specific IgG for different time points. Differences between the groups were assessed using the Wilcoxon test with Holm’s correction for multiple testing. The strength of correlations was assessed by Spearman’s correlation test. *p < 0.05, **p < 0.01, ***p < 0.001, ****p < 0.0001.

We, therefore, measured the titers of S1-specific IgG and IgA with an ultrasensitive ELISA in saliva. Our findings demonstrate that S1-specific IgG levels in saliva decrease by 2.5-fold between months 3 and 6 after full vaccination (P<0.0001). Following booster vaccination in month 9, the S1-specific IgG levels increased 8.4-fold on month 12 when compared to month 6 (P<0.0001) (**Figure 2A**). In contrast, the S1-specific IgA titer remained relatively stable during the entire monitoring with a subtle 1.3-fold increase between months 6 and 12 (P<0.001) (**Figure 2B**). Since we used the same monoclonal antibody with a variable Fc region as a calibrator for the IgG and IgA ELISAs, we were able to compare the relative amounts of both antibody isotypes. Interestingly, the proportion of S1-binding IgA was significantly increased at month 6 when compared to month 3 (P<0.05), suggesting that IgA persists longer in saliva than IgG. At month 12 IgG was the predominant isotype of salivary antibodies recognizing the S1-domain of the spike protein. Its proportion was significantly higher than at months 3 and 6 (P<0.01 and P<0.0001, respectively) (**Figure 2C**). The levels of S1-specific IgA and IgG in saliva correlated for all time points (**Figure 2D**), as did the levels of S1-specific IgG in saliva and plasma (**Figure 2E**).

Collectively, these data demonstrate a decrease of SARS-CoV-2-specific antibodies in saliva with time after full vaccination and the resurge of mostly IgG antibodies following booster vaccination.

### The frequency of SARS-CoV-2-specific memory B cells increases over time and with booster vaccination

Studies have shown that SARS-CoV-2-specific memory B cells remain at elevated levels for a much longer time after the infection or vaccination than the antibodies making them particularly important for long-term immunological memory (25, 29).

**Figure 3:**
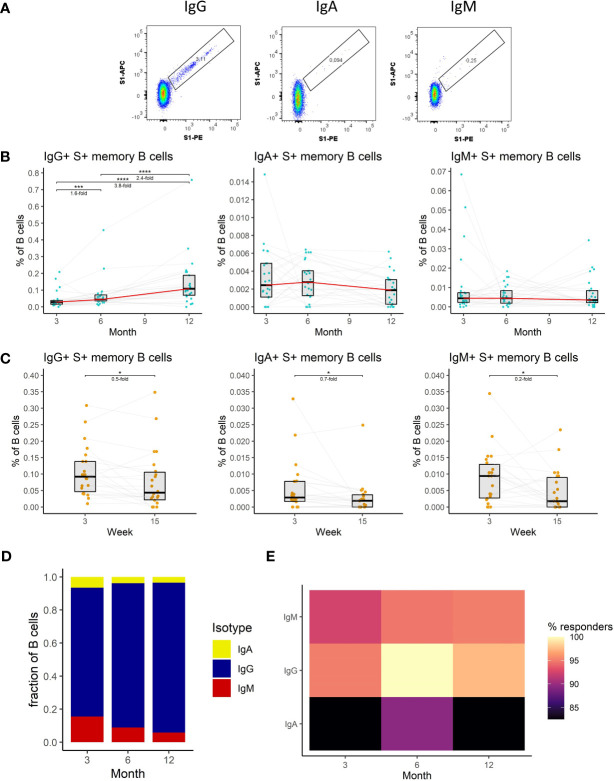
The dynamics of SARS-CoV-2-specific memory B cell response after the initial and booster doses of mRNA vaccine. **(A)** Representative flow cytometry plots for identification of S1-specific memory B cells with different BCRs. **(B)** Frequencies of IgG+, IgA+, and IgM+ S1-specific memory B cells as a percentage of total B cells in the peripheral blood of vaccinated individuals for months 3, 6, and 12 after the initial vaccination. The red line connects the median values of each time point. Fold change was calculated as a ratio of the medians of compared time points. **(C)** Frequencies of IgG+, IgA+, and IgM+ S1-specific memory B cells in peripheral blood of vaccinated individuals 3 to 15 weeks after the booster vaccination. **(D)** Relative proportions of S1-specific memory B cells bearing BCRs of a different isotype. **(E)** Percentage of individuals with detectable S1-specific memory B cells according to the BCR isotype and time point. Differences between the groups were assessed using the Wilcoxon test with Holm’s correction for multiple testing.

We, therefore, measured the frequency of S1-specific memory B cells in the peripheral blood of vaccinated individuals at each of the sampling time points utilizing multiparameter flow cytometry. We distinguished S1-specific memory B cells according to their B cell receptor (BCR) isotype; IgA+, IgM+, IgG+ (**Figure 3A**) (detailed gating strategy is available in **Supplemental figure 1**). Unlike in the case of antibodies the frequency of IgG+ S1-specific memory B cells increased between months 3 and 6 by 1.6-fold (P<0.001) and further rose 2.4-fold (P<0.0001) between months 6 and 12 (**Figure 3B**). The frequency of these cells only decreased immediately following the recall response evoked by the booster vaccination; 2-fold decrease between weeks 3 and 15 following the booster (P<0.05) (**Figure 3C**). The frequencies of IgA+ and IgM+ S1-specific memory B cells remained stable between months 3, 6, and 12 (**Figure 3B**), but also decreased between the third and fifteenth week following the booster vaccination (1.4-fold, P<0.05, and 5-fold, P<0.05 respectively) (**Figure 3C**). Next, we compared the relative frequencies of S1-specific memory B cells according to their BCR isotype for months 3, 6, and 12 after the full vaccination. Of note, the frequencies of S1-specific IgA+ and IgM+ memory B cells were much lower than those of IgG+ cells. Their proportions were highest at month 3 and then decreased with each of the following time points (**Figure 3D**). The percentages of individuals with detectable S1-specific memory B cells were generally high. Most of the individuals had detectable IgG+ S1-specific memory B cells followed by IgM+, individuals with IgA+ cells were rare (**Figure 3E**).

To sum up, we have shown that IgG+ memory B cells dominate the SARS-CoV-2-specific B cell response. The frequency of these cells kept increasing with time. Following the booster vaccination, their frequency initially dropped but remained elevated compared to the initial 2-dose immunization.

### SARS-CoV-2-specific CD4+ T cell responses are durable and moderately augmented by booster vaccination

CD4+ T cells are a key component of a vaccine-induced immune response since they regulate antibody production by B cells (30, 31). Besides their stimulatory and coordinating functions, these cells can also act cytotoxic and directly kill infected cells (32).

**Figure 4:**
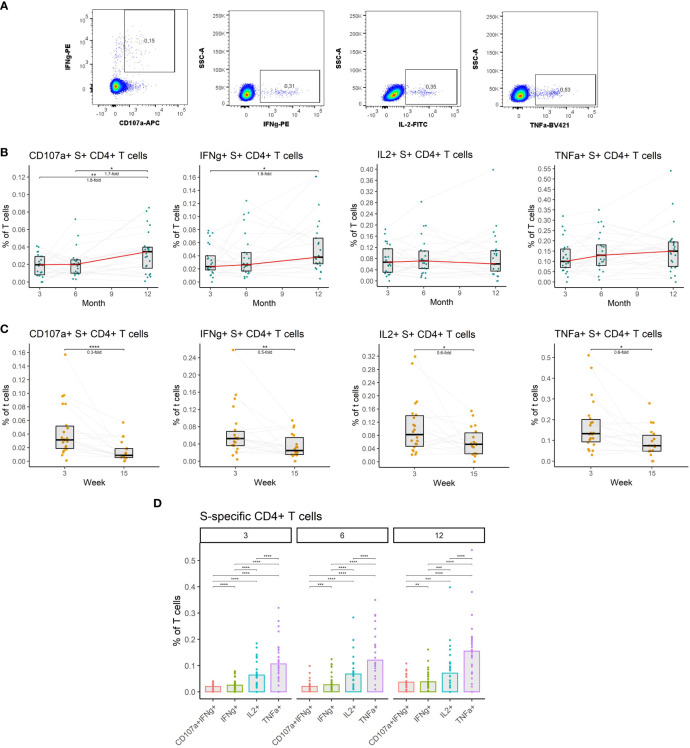
The dynamics of SARS-CoV-2-specific CD4+ T cells after the initial and booster doses of mRNA vaccine. **(A)** Representative flow cytometry plots demonstrating the detection of S-specific CD4+ T cells with different effector functions; cytotoxicity (CD107a and IFNγ expression), IFNγ expression, IL-2 expression, and TNFα expression. **(B)** The frequencies of S-specific CD4+ T cells with different effector functions as a percentage of bulk T cells for months 3, 6, and 12 after the initial vaccination. The red line connects the median values of each time point. Fold change was calculated as a ratio of the medians of compared time points. **(C)** The frequencies of S-specific CD4+ T cells with different effector functions in the peripheral blood of vaccinated individuals 3 to 15 weeks after the booster vaccination. **(D)** Comparison of S-specific CD4+ T cell frequencies with different functions for months 3, 6, and 12 after the initial vaccination. Differences between the groups were assessed using the Wilcoxon test with Holm’s correction for multiple testing. *p<0.05, **p<0.01, ***p<0.001, ****p<0.0001.

Given their importance, we measured the frequencies of CD4+ T cells specific for the spike (S) protein of the SARS-CoV-2 in the peripheral blood of vaccinated individuals. Antigen-specific T cells were detected by peptide stimulation and subsequent detection of cytokine expression by multiparameter flow cytometry. Four major functions of the CD4+ T cells were monitored: cytotoxicity (CD107a and IFNγ expression), IFNγ expression, IL-2 expression, and TNFα expression (**Figure 4A**) (detailed gating strategy is available in **Supplemental figure 2**). The frequency of S-specific CD4+ T cells remained stable between months 3 and 6 after the full vaccination regardless of the function. After the booster vaccination on month 9, the frequency of S-specific cytotoxic CD4+ T cells rose by 1.7-fold (P<0.05), and the frequency of IFNγ-expressing CD4+ T cells rose by 1.6-fold (P<0.05) between the months 6 and 12. Frequencies of IL-2- and TNFα-expressing CD4+ T cells did not significantly change during this period (**Figure 4B**). Between weeks 3 and 15 following booster vaccination we observed a decline in S-specific CD4+ T cell frequencies. The frequency of cytotoxic cells decreased 3.3-fold (P<0.0001), the frequency of IFNγ-expressing cells 2-fold (P<0.01), the frequency of IL-2-expressing cells 1.7-fold (P<0.05) and the frequency of TNFα-expressing cells 1.7-fold (P<0.05) (**Figure 4C**). TNFα expression was the most frequent function among the S-specific CD4+ T cells followed by IL-2 expression, IFNγ expression, and cytotoxicity. This was true for months 3, 6, and 12 (**Figure 4D**).

Collectively, these findings indicate that vaccination induces a durable SARS-CoV-2-specific CD4+ T cell response that is moderately augmented by the booster vaccination.

### SARS-CoV-2-specific CD8+ T cell responses are durable and moderately augmented by booster vaccination

CD8+ T cells can recognize and kill infected cells and thus represent an important antiviral mechanism. Studies have shown that SARS-CoV-2-specific CD8+ T cells successfully limit the infection and positively correlate with protection from severe disease (33, 34).

**Figure 5:**
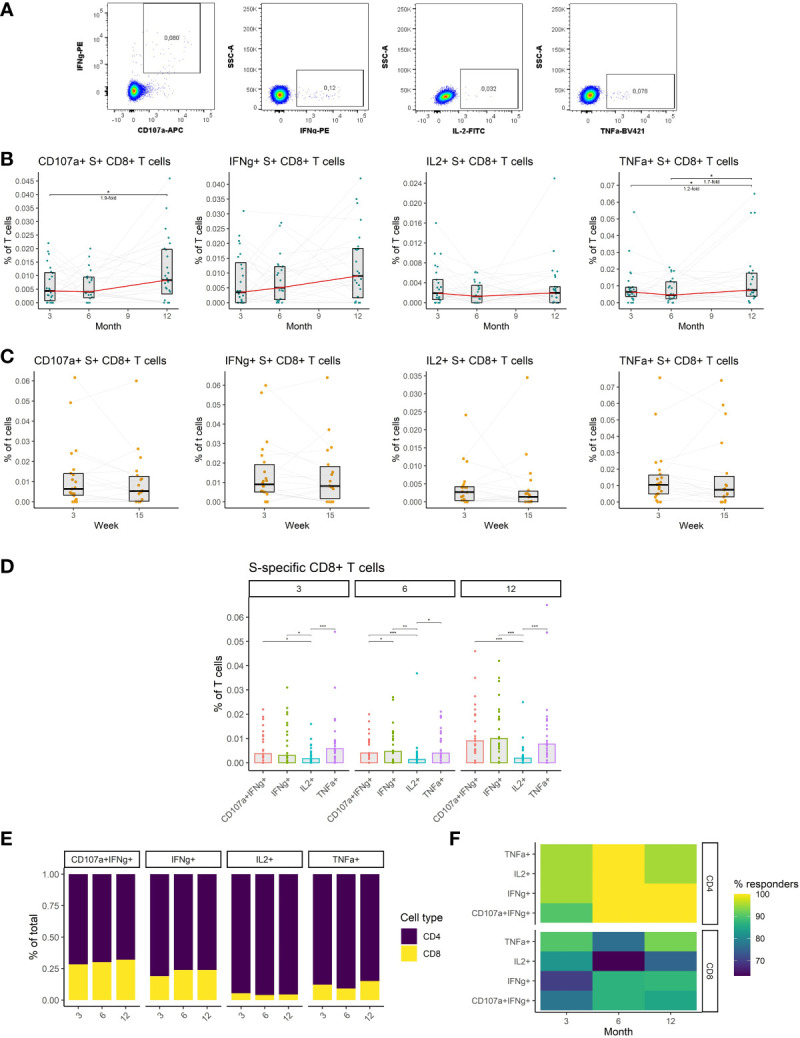
The dynamics of SARS-CoV-2-specific CD8+ T cells after the initial and booster doses of mRNA vaccine. **(A)** Representative flow cytometry plots demonstrating the detection of S-specific CD8+ T cells with different effector functions; cytotoxicity (CD107a and IFNγ expression), IFNγ expression, IL-2 expression, and TNFα expression. **(B)** The frequencies of S-specific CD8+ T cells with different effector functions as a percentage of bulk T cells for months 3, 6, and 12 after the initial vaccination. The red line connects the median values of each time point. Fold change was calculated as a ratio of the medians of compared time points. **(C)** The frequencies of S-specific CD8+ T cells with different effector functions in the peripheral blood of vaccinated individuals 3 to 15 weeks after the booster vaccination. **(D)** Comparison of S-specific CD8+ T cell frequencies with different functions for months 3, 6, and 12 after the initial vaccination. **(E)** Relative proportions of CD4+ and CD8+ S-specific T cells with different effector functions for months 3, 6, and 12 after the initial vaccination. **(F)** Percentage of individuals with detectable S1-specific CD4+ or CD8+ T cells according to the effector function and time point. Differences between the groups were assessed using the Wilcoxon test with Holm’s correction for multiple testing. *p < 0.05, **p < 0.01, ***p < 0.001, ****p < 0.0001.

We, therefore, investigated S-specific CD8+ T cell responses in the peripheral blood of vaccinated individuals. Antigen-specific cells were identified by peptide stimulation and flow-cytometric detection of effector molecules (**Figure 5A**) (detailed gating strategy is available in **Supplemental figure 2**) as in the case of CD4+ T cells. The data revealed increased frequencies of cytotoxic and TNFα-expressing S-specific CD8+ T cells at month 12 after vaccination. The frequency of cytotoxic cells increased by 1.9-fold (P<0.05) between months 3 and 12, while the frequency of TNFα-expressing cells increased 1.3-fold (P<0.05) during the same period. No significant changes were observed between months 3 and 6 for any of the assessed functions (**Figure 5B**). Unlike the rest of the immune responses, the frequency of S-specific CD8+ T cells did not decline between weeks 3 and 15 after the booster vaccination, for any of the functions, suggesting the persistence of these cells (**Figure 5C**). The frequencies of cytotoxic, IFNγ- and TNFα-expressing S-specific CD8+ T cells did not change over time, while the frequency of IL2-expressing cells trended to be lower (**Figure 5D**). Comparing CD8+ and CD4+ S-specific T cell responses, CD4+ T cells were generally more frequent than CD8+ T cells. The highest proportion of CD8+ T cells was observed among the cytotoxic followed by IFNγ-expressing, TNFα-expressing, and IL2-expressing S-specific T cells. No significant differences were observed between the three time points for any of the functions (**Figure 5E**). Similarly, the percentage of individuals with detectable S-specific T cells was higher in the case of CD4+ T cells for all functions and at all three time points (**Figure 5F**).

Taken together, we have demonstrated that mRNA vaccines induce SARS-CoV-2-specific CD8+ T cells that remain stable after initial and booster vaccinations. These cells were considerably less frequent than SARS-CoV-2-specific CD4+ T cells.

## Discussion

There is increasing evidence that SARS-CoV-2 vaccination mounts a robust adaptive immune response (1–4), however, similar to infection-induced immunity, the longevity of this response is limited (35, 36). In addition to the ongoing emergence of new variants, this is one of the primary reasons behind the increasing infection incidence despite the high seropositivity of the population. Here we longitudinally assessed the adaptive immune response to the initial 2 doses but also the third booster dose of mRNA vaccine in healthy SARS-CoV-2 naïve individuals. Our data demonstrate a decline in the antibody response after the initial vaccination. At the same time, memory B cell and T cell frequencies proved to be more stable or even increased in magnitude. Importantly, booster vaccination significantly improved humoral and cellular responses compared to the initial two doses of mRNA vaccine. Nevertheless, most immune responses decreased with time after the booster dose.

Neutralizing antibodies are generally the main antiviral mechanism induced by vaccination (37) and the assessment of SARS-CoV-2 immunity most often relies on the measurement of spike-specific antibodies in plasma. In concordance with previous studies (35, 36, 38), we have demonstrated that the neutralizing antibody titer rapidly declines after the initial immunization with 2 doses of mRNA vaccine as well as after the booster vaccination. The booster vaccination remarkably augmented the neutralizing antibody levels when compared to the initial vaccination (39). Of note, the most profound boosting effect was observed for the omicron variant which has generally been more resistant to neutralization than alpha and delta variants. Apart from increasing the overall spike-specific and neutralizing antibody titer booster dose also increased the neutralizing potency of these antibodies against the delta and omicron variants. Improved potency and breath of SARS-CoV-2-neutralizing antibodies after booster vaccination has also been previously observed and has been attributed to the increased proportion of B cell clones targeting conserved regions of the receptor-binding domain (40, 41). Furthermore, spike-specific antibody levels correlated with plasma neutralization capacity for all variants and time points indicating that spike-specific antibody titer is a good surrogate of neutralization. Interestingly, the correlations at 6 months after vaccination showed the best association between the antibody titer and neutralization suggesting maturation of the antibody response with time after vaccination. Collectively, these findings demonstrate a rapid waning of antibody response and emphasize the importance of booster vaccination.

Apart from plasma, antibodies can also be found in other body fluids including saliva (42). This might be particularly important since SARS-CoV-2 initially infects the upper respiratory tract (28). Our findings and those of others show that 2 doses of mRNA vaccine successfully elicited spike-specific antibodies in saliva (43, 44). Similar to those found in plasma, their titer rapidly declined with time as previously documented (43), and got augmented by booster vaccination. Interestingly, IgA antibodies showed higher stability over time than IgG but were less abundant, especially early after the vaccination. The levels of both isotypes correlated for all time points indicating coordinated production. Moreover, the levels of spike-specific IgG in saliva correlated with the plasma levels of these antibodies for all time points, suggesting that salivary anti-spike IgG partially originate from the plasma (42). To sum up, antibodies in saliva generally followed the same kinetics as those in plasma, however, IgA showed increased stability and might be important for long-term protection from infection.

Memory B cells have previously been identified as a particularly persistent component of immunity elicited by SARS-CoV-2 infection (25, 29). Similar to infection, also vaccination triggers the buildup of SARS-CoV-2 spike-specific memory B cells (7, 45). In contrast to antibodies, however, their frequencies increase for several months after vaccination (29). A similar trend was observed for spike-specific IgG+ but not IgM+ and IgA+ memory B cells following the initial two doses of mRNA vaccine. While there was a decline in the frequency of spike-specific memory B cells immediately after booster vaccination, which is probably due to the downregulation of the immune response following antigen clearance, their levels increased compared to the initial vaccination, as previously reported by others (46). IgG was the predominant BCR isotype for all time points as previously observed for individuals recovered from infection (25), and its proportion kept increasing over time. Although much less frequent than IgG, IgM-bearing spike-specific memory B cells were more abundant than those with IgA BCR. Moreover, spike-specific IgA+ memory B cells were not detectable in the largest proportion of individuals for all time points. Collectively, IgG+ spike-specific memory B cell frequencies increase for at least 6 months after the initial vaccination and further expand after the booster vaccination making them a key component of long-term SARS-CoV-2 immunity.

To efficiently generate an antibody response a vaccine must also trigger the formation of virus-specific CD4+ T cells that provide B cells with signals crucial for the production of high-affinity antibodies. Studies have demonstrated that mRNA vaccines successfully induce CD4+ T cell responses (8, 30). We have demonstrated that the frequencies of spike-specific CD4+ T cells with different functions (cytotoxicity, IFNγ expression, IL-2 expression, and TNFα expression) remained relatively stable for at least 6 months after the initial 2 doses of vaccine. Similar to B cells, their frequencies rapidly declined between 3 and 15 weeks following booster vaccination. Compared to the initial vaccination, the booster dose moderately increased frequencies of cytotoxic and IFNγ-expressing cells but did not affect the cells with other functions as previously observed (47). TNFα expression was the most prevalent function among the spike-specific CD4+ T cells followed by IL-2 expression, IFNγ expression, and cytotoxicity. Taken together these findings suggest the persistence of SARS-CoV-2 spike-specific CD4+ T cells following initial vaccination and the moderate effect of booster vaccination on these cells.

It has been previously documented that mRNA vaccines induce the formation of CD8+ T cell response (48–50). Similar to CD4+ T cells, we have observed that CD8+ T cell frequencies remain relatively stable after the initial immunization. In contrast to other immune responses, no decrease in the frequency of these cells was observed between weeks 3 and 15 following booster vaccination. In line with previous studies booster dose only moderately increased the frequencies of cytotoxic and TNFα-expressing cells compared to the initial immunization (48). Most of the spike-specific CD8+ T cells were either cytotoxic, expressed IFNγ or TNFα. IL-2-expressing cells were rare. The observed differences in the functional profiles of CD4+ and CD8+ T cells are most likely due to the different biologies of the two cell kinds; CD8 T cells are less capable of IL-2 production than CD4s (51). Considering the ratio between CD4+ and CD8+ T cells among the spike-specific T cells, the latter were notably less abundant for all time points. Furthermore, a larger proportion of individuals lacked spike-specific CD8+ than CD4+ T cell response. This can be explained by the design of mRNA vaccines that primarily targets the production of antibodies and not cytotoxic T cells. Altogether, SARS-CoV-2–specific T cell frequencies were stable after the initial 2-dose vaccination and mildly increased after the booster vaccination. Of note, CD4+ T cells were more abundant than CD8+ T cells.

In the present study, we have assessed the adaptive immune response to the mRNA vaccines after the initial and booster immunizations in healthy SARS-CoV-2 naïve individuals. We showed that antibody titer decreases rapidly after the initial 2 doses but is augmented following the booster vaccination. Booster vaccination was particularly important for the neutralization of the currently circulating omicron variant. Similar kinetics were observed for the salivary antibodies, with exception of IgA whose levels were relatively stable. The memory B cells and T cells showed to be more durable than the antibodies and were also positively affected by booster vaccination, making them particularly important for durable protection against severe SARS-CoV-2 infection.

## Data availability statement

The original contributions presented in the study are included in the article/**Supplementary Material**. Further inquiries can be directed to the corresponding author.

## Ethics statement

The studies involving human participants were reviewed and approved by Ethics Committee of the Medical Faculty of the University of Bonn (ethics approval numbers 125/21). Written informed consent to participate in this study was provided by the participants’ legal guardian/next of kin.

## Author contributions

Conceptualization, JP, WP. Methodology, JP. Investigation, JP, WP, KP, JZ, MB, CS, HP. Resources, JP, WP. Writing-Original Draft, JP, WP. Writing-Review & Editing, HS. Funding acquisition, JP, HS. Supervision, JP, HS. All authors contributed to the article and approved the submitted version.
