# Supplementary figures and images for: Longitudinal monitoring of mRNA-vaccine-induced immunity against SARS-CoV-2
Source: Front Immunol. 2023 Jan 19;14:1066123. doi: 10.3389/fimmu.2023.1066123 (PMC9893859; doi:10.3389/fimmu.2023.1066123)

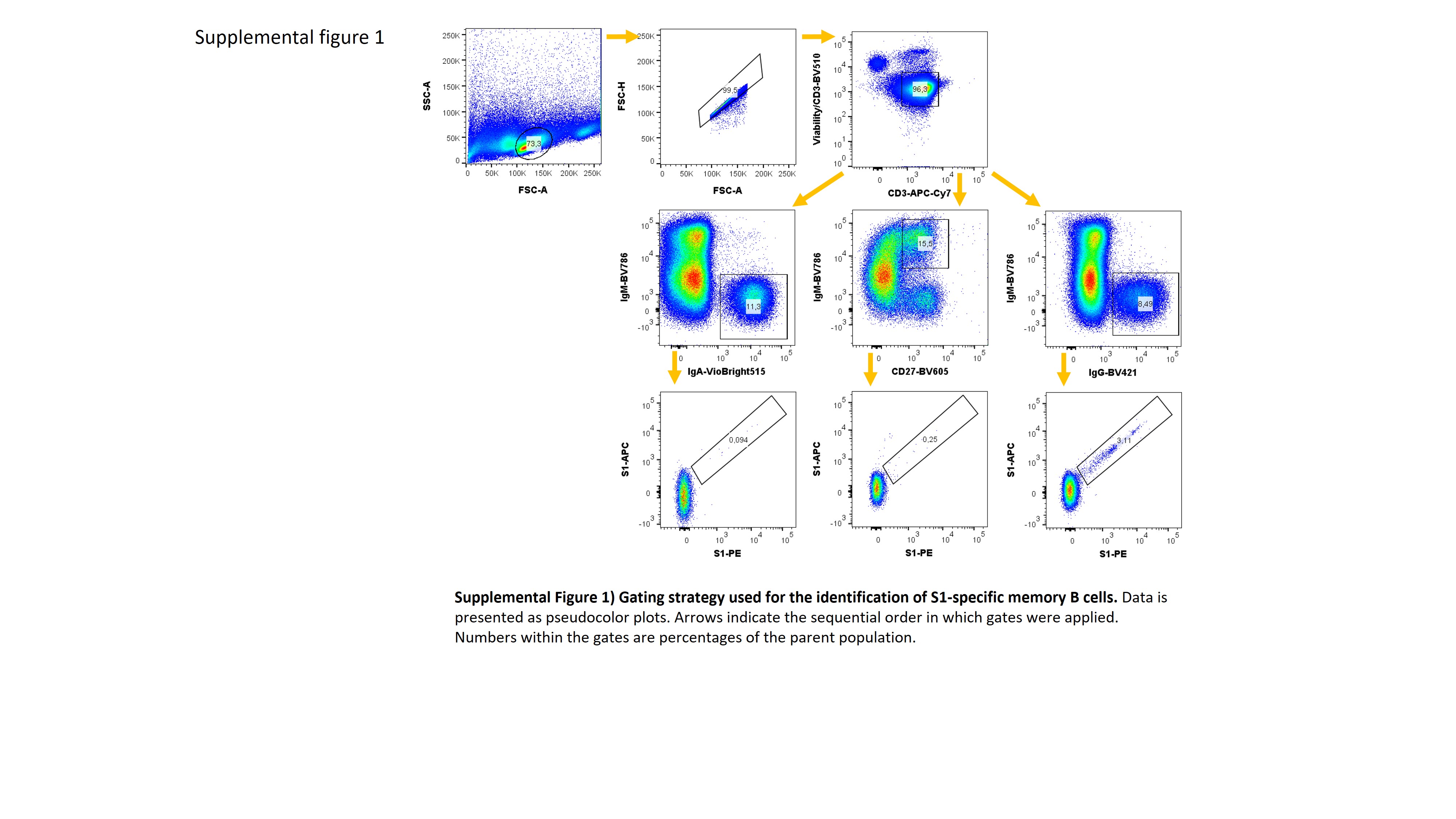

Supplement: Supplementary file 1 [file Image_1.jpeg]

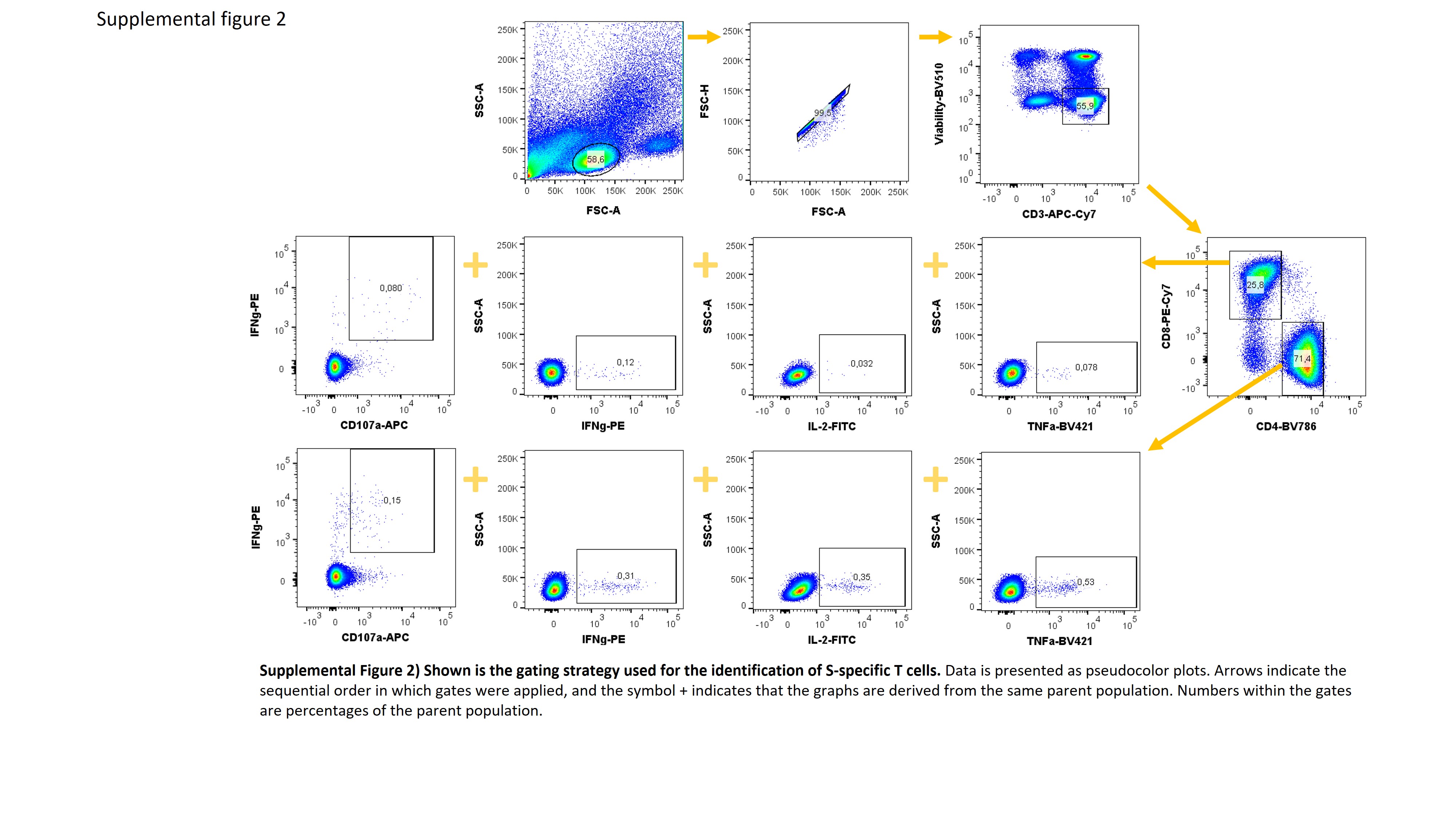

Supplement: Supplementary file 2 [file Image_2.jpeg]
